# Changes of depression and job stress in workers after merger without downsizing

**DOI:** 10.1186/s40557-018-0266-4

**Published:** 2018-08-29

**Authors:** Jun Ick Jung, Jun Seok Son, Young Ouk Kim, Chang Ho Chae, Chan Woo Kim, Hyoung Ouk Park, Jun Ho Lee, Young Hoo Shin, Jea Chul Ha

**Affiliations:** Department of Occupational and Environmental Medicine, Samsung Changwon hospital, Sungkyunkwan University School of Medicine, 158, Paryong-ro, Masanhoewon-gu, Changwon-si, Gyeongsangnam-do 51353 Republic of Korea

**Keywords:** Restructuring, Depression, Job stress

## Abstract

**Background:**

Since the 1980s, restructuring, which includes downsizing, closures, mergers, and privatization, has expanded worldwide, and various studies have investigated its effect on health. However, previous studies have mainly focused on restructuring accompanied by massive lay-offs, and the effect of a merger on workers’ health is still controversial. This study aims to investigate changes in worker depression and job stress after a merger without downsizing, which is unusual in Korea.

**Methods:**

Repeated surveys were done in April 2014, April 2015, and April 2016 involving the participation of 209 subjects. Participants were divided into two groups, which were comprised of blue-collar workers (104) and white-collar workers (105). Sociodemographic characteristics, including age, education level, job tenure, gender, marital status, smoking status, and alcohol consumption, were measured via a survey. To determine the level of depression, the Korean version of the Center for Epidemiologic Studies Depression Scale (CES-D) was employed, and to investigate job stress, the Korean Occupational Stress Scale-Short Form (KOSS-SF) was used. For statistical analyses, Pearson’s chi-square test, the Student’s t-test, and repeated measure analysis of variance (ANOVA) were performed.

**Results:**

The results showed that depression (CES-D, F[2, 400] = 0.466, *p* = 0.628) was changed but without significance and job stress (KOSS-SF, F[1.899, 379.831] = 3.192, *p* = 0.045) were significantly different. The between-group difference in the CES-D score between the blue- and white-collar workers by survey administration time was not statistically significant (F = 0.316, *p* = 0.574). The interaction between the survey time and occupational group was also not statistically significant (F = 0.967, *p* = 0.381). The between-group difference in the KOSS-SF total score was not statistically significant (F = 1.132, *p* = 0.289), and the interaction between the survey administration time and occupational group was also not significant (F = 0.817, *p* = 0.437). In the job stress subgroup analyses Job insecurity and Lack of reward showed a significant difference by survey administration time.

**Conclusion:**

This study showed that a merger without massive downsizing can cause negative health effects such as an changes in depression and increase in job stress. To improve the health of workers, both the immediate negative effects on health, and the long-term effects or their resolution over time should be considered prior to the merger.

## Background

Restructuring began in the early 1980s owing to globalization and the pursuit of efficiency. Beginning with the “Next Step,” which was a civil service restructuring program of Thatcherism in the UK in 1988, restructuring has expanded its scope with no discrimination between public and market domains. Unlimited competition has become a key survival issue for companies owing to the loss of global control function and unpredictable market circumstances, which has inevitably forced the adoption of restructuring strategies [1]. Restructuring is defined as implementing changes that target not only specific organizations, but the entire scope of an enterprise. Changes include downsizing, closures, mergers, and privatization, but mostly involve downsizing [2]. Downsizing due to restructuring is different from the traditional concept of lay-offs. In contrast with lay-offs in the past that targeted manufacturing and production industry workers, downsizing due to restructuring targets executives, experts, and white-collar workers, of whom the proportion of male workers with a high education level has increased [3]. Because of the psychosocial impact effected, not only on victims of restructuring but also on survivors, studies into the health effects they experience have been proposed. Accordingly, studies have found that workers who were subject to lay-offs showed a mixed state of behavioral and emotional patterns such that a layoff survivor syndrome has emerged, which is defined as demotivation, cynicism, anxiety, low morale, and reduction in dedication to the organization [4]. In Korea, because restructuring after the International Monetary Fund (IMF) Korean Financial Crisis in 1997 was followed by mass lay-offs and the expansion of temporary workers, significant attention has been paid to the effect of restructuring [5].

Restructuring has expanded throughout society, affecting the health of workers and prompting several studies. In the biological and physical domains, restructuring has been known to affect cardiovascular mortality [6], blood pressure [7], immunoglobulin G, estradiol, apolipoprotein A1 [8], dehydroepiandrosterone sulphate (DHEAS), cortisol [9], and musculoskeletal pain [10]. In the mental health domain, psychosocial factors have been known to degrade the health level, quality of life, and productivity of workers in relation to neurosis or distress, which has increased the social burden accordingly [11, 12]. Previous studies have primarily targeted the type of restructuring that is accompanied by lay-offs. The health effects on victims and survivors were reported as increased certified sickness absence [13], increased medically certified musculoskeletal sickness absence [10], decreased self-rated health [14], decreased satisfaction and job security [15], increased incidence of mental health problems [16], increased depression [17], and disrupted well-being [18]. Some of the studies on restructuring were conducted with regard to the merging of companies, but the effects on health examined by these studies were not disclosed as comprehensively as for the case of lay-offs. One study on mergers accompanied by lay-offs reported increased anxiety and impatience [19], whereas another reported no change in negative emotions [20]. Some studies on mergers without lay-offs reported meaningful effects, such as a reduction in job satisfaction [21], deterioration in quality of life, and increased job stress [22], whereas others reported no significant differences in depression [23] or psychiatric events [24], providing conflicting results.

Because adverse effects due to restructuring may be sustained only over the short-term, or over several years in the long-term [25], it is important to identify the variables that may have an impact on health effects in order to establish management strategies during restructuring, or to reduce the individual and societal burden through work-related stress intervention [26]. Some studies [27, 28] reported that the degree of downsizing was quite important for determining the extent of negative health effects, but few previous studies have been conducted on decisive factors in the case of a merger without downsizing. However, Väänänen et al. (2011) [24] reported that non-manual labor workers who experienced negative changes had a doubled psychiatric event risk, whereas no changes were found in manual labor workers, which implied that differences in health effects due to a merger existed between traditional occupational groups for blue and white-collar workers.

Interestingly, a merger of a manufacturing factory in Gyeongsangnam-do was announced in November 2014, and the merger process was completed in June 2015. During this process, no systematic downsizing was conducted (a total of six workers were laid off). This study aimed to determine the effect of restructuring due to a merger without downsizing, which is unusual in Korea, on workers’ mental health and job stress. A survey with a scaled tool was used to determine whether significant changes had taken place between the different times that the survey was conducted, and to verify whether the difference between blue- and white-collar occupational groups was a significant variable that affected the health of workers due to a merger.

## Methods

### Subject

This study was conducted on workers in a single manufacturing business. Workers had their health status checked at a hospital in Gyeongsangnam-do. This defense industry company has approximately 1800 employees and assembles aero-space engine component. A sudden merger of their workplace, which was one of the affiliates in a conglomerate, was announced without prior notice on November 26, 2014 to the rest of the conglomerate. This sudden announcement had no concrete plans of merger, there were no confirmation of succession of employment and followed merger process, detailed informations about merger have never been provided to employees. During these unclear periods a worker’s union was founded in December 2014. The company name was changed in a shareholders’ meeting on June 29, 2015. During this process, only six workers were laid off, one worker was placed on an unlimited suspension, and 53 workers experienced disadvantages due to work suspension or pay cuts. However, the merger process was completed without a massive downsizing. The survey was conducted three times: 1148 employees had a health check-up in 2014 but this study was designed to longitudinal observation with same time range only workers surveyed in April 2014 were recruited. So in First survey at April 2014, 371 workers were participated, the second survey was conducted in April 2015, and the third survey was conducted in April 2016. As 71 workers had had their health status checked at an undesignated time, missed their results, or did not respond, only 300 workers completed the second survey. For the third survey, 209 workers were selected as the final subjects, excluding 91 respondents of the second survey. The blue- and white-collar workers were differentiated by their registered information. After classifying the workers, 104 blue-collar and 105 white-collar workers comprised the final subjects. This study was reviewed by the institutional review board (IRB No. 2018–04-001) before implementation.

### General characteristics

In the survey, general sociodemographic characteristics such as age, education level, job tenure, gender, and marital status, which are known to affect job stress [29], and alcohol consumption, which is known to affect job stress [30], were measured via survey. The education level characteristic was divided into a high school graduation or lower group, and a college graduation or higher group. The marital status characteristic was divided into not married and married. Alcohol drinking habits followed the high-risk drinking classification criteria set forth in the Korea National Health and Nutrition Examination Survey [31]. That is, if a worker had a drink more than twice a week and had seven glasses a day for men or five glasses a day for women, he/she was classified as belonging in the high-risk drinking group.

### Depression

To determine the level of depression, the Korean version of the Center for Epidemiologic Studies Depression Scale (CES-D) [32], a self-report type scale developed by the National Institute of Mental Health (NIMH) in the USA, was employed. The CES-D is a widely used tool that distinguishes depressive symptoms from the general population and is known to be valid for differentiating between those who have depressive symptoms and those who do not. The CES-D consists of 20 questions for a total score of 60 points. The scale was divided into four levels, from zero to three points, which related to the frequency of depressive symptoms experienced during the previous week. The higher the score, the worse the depression status.

### Job stress

To determine the level of job stress, the Korean Occupational Stress Scale-Short Form (KOSS-SF) [33] was employed to find general, but Korean-specific job stress factors. The KOSS-SF consists of 24 questions in seven categories: job demand, insufficient job control, interpersonal conflict, job insecurity, organizational system, lack of reward, and occupational climate. These categories are borrowed from the eight basic categories of the Korean Occupational Stress scale (KOSS), including the physical environment category, which was excluded from the KOSS-SF due to its tendency toward specific occupation-related questions through the national scale samples. A converted score is calculated for each category, and a total sum of all the converted scores in all categories becomes the total score, which is then divided by the number of categories. The higher the score, the more severe the job stress. In addition, the KOSS-SF presents a reference value that can evaluate the level of job stress with regard to the total score of the KOSS-SF and sub-scores in the seven categories according to gender by quartile.

### Statistical analysis

Pearson’s chi-square test and Student’s t-test were employed to analyze the sociodemographic characteristics of the subjects. A repeated measure analysis of variance (ANOVA) was conducted to determine the changes in mental health and job stress before and after the merger, and again 1 year later. Finally, a repeated measures ANOVA was conducted again to analyze the differences in mental health and job stress over time and determine whether the survey time and occupational group were interactive in terms of mental health and job stress. All statistical analyses were conducted using SPSS Version 21 (IBM Corp., Armonk, NY, USA), and the statistical level of significance was set to 0.05.

## Results

### Subject characteristics

The total number of study subjects was 209, and their sociodemographic characteristics were as follows. The mean age was 26.52 ± 3.78 years, the mean tenure was 6.22 ± 3.87 years, and there were 172 male workers (82.3%) and 37 female workers (17.7%). The education level indicated that 110 workers had a high school education or less (52.6%), and 149 workers were married (71.3%). The number of non-smokers was 133 (63.6%), which was the majority, and the number of people in the high-risk drinking group was 87 (41.6%).

In terms of occupational groups, there were 104 blue-collar workers (49.76%) and 105 white-collar workers (50.24%). The baseline CES-D score and KOSS-SF score measured at the first survey administration, and the sociodemographic characteristics between the two groups are as follows (Table 1). The mean ages of the blue and white-collar workers were 25.94 ± 4.56 and 27.10 ± 2.71 years, respectively, which was a statistically significant difference (*p* = 0.027). The job tenures of the blue- and white-collar workers were 7.64 ± 3.66 and 4.81 ± 3.55 years, respectively, which showed that the job tenure of the blue-collar workers was significantly higher statistically (*p* < 0.001). The male-to-female gender ratio was 98:6 in the blue-collar worker group and 74:31 in the white-collar worker group, which represented a significant difference between the two (*p* < 0.001). The ratio of the education level (high school graduates or lower to college graduates or higher) was 84:20 in the blue-collar worker group and 26:79 in the white-collar worker group, which showed a significant difference (*p* < 0.001). The marital status ratio (non-married to married) was 30:74 in the blue-collar worker group and 30:75 in the white-collar worker group, which showed a no statistically significant difference (*p* = 0.965). The blue-collar worker group had 47 smokers, while the white-collar worker group had 29 smokers, which showed that the blue-collar worker group had significantly more smokers than the white-collar worker group (*p* = 0.008). Similarly, the blue-collar worker group had more high-risk drinkers (53 workers) than the white-collar worker group (34 workers), which was significantly different statistically (*p* = 0.006).

**Table 1:** General characteristics, baseline CES-D score, KOSS-SF total score differences between subject groups in 1st survey (April 2014)

Variables	Total (*n* = 209) Mean ± SD or N	Non-office worker (*n* = 104) Mean ± SD or N (%)	Office-worker (*n* = 105) Mean ± SD or N (%)	*P* value^*^
Age, years	26.52 ± 3.78	25.94 ± 4.56	27.10 ± 2.71	0.027
Tenure, years	6.22 ± 3.87	7.64 ± 3.66	4.81 ± 3.55	< 0.001
CES-D^a^ score	7.75 ± 7.94	8.20 ± 7.39	7.30 ± 8.46	0.411
KOSS-SF^b^ score	45.03 ± 12.23	46.18 ± 11.32	43.89 ± 13.02	0.176
Gender (Male/Female)	172(82.3)/37(17.7)	98(94.2)/6(5.8)	74(70.5)/31(29.5)	< 0.001
Educational level (≤High school/≥College)	110(52.6)/99(47.4)	84(80.8)/20(19.2)	26(24.8)/79(75.2)	< 0.001
Marital status (Unmarried/Married)	60(28.7)/149(71.3)	30(28.8)/74(71.2)	30(28.6)/75(71.4)	0.965
Smoking status (Never/Ex-, current)	133(63.6)/76(36.4)	57(54.8)/47(45.2)	76(72.4)/29(27.6)	0.008
Alcohol intake (Non-risky/Risky)	122(58.4)/87(41.6)	51(49.0)/53(51.0)	71(67.6)/34(32.4)	0.006

*SD* Standard deviation, *N* number

* *P* value was calculated by Student’s t-test or Pearson’s chi-square test between Non-office worker and office-worker group

^a^ CES-D The center for epidemiologic studies depression scale

^b^ KOSS-SF: Korean Occupational Stress Scale-Short Form (KOSS-SF)

### Analysis of the CES-D and KOSS-SF scores

The means and standard deviations of the CES-D score and KOSS-SF total scores measured in the first, second, and third surveys are given in Table 2. To determine whether the change in survey results was significant based on the survey administration period, a repeated measures ANOVA was performed. The results showed that the difference between the depression level (F[2, 416] = 9.255, *p* < 0.001) and job stress total score (F[1.872, 389.460] = 40.195, *p* < 0.001) was statistically significant. The results of the post-hoc analysis with Bonferroni correction showed that the CES-D score increased significantly between the first and second surveys (*p* < 0.001), and then decreased significantly between the second and third surveys (*p* = 0.033). The score remained high (*p* = 0.192) although it was not statistically significant compared to the first survey result. The KOSS-SF total score increased significantly between the first and second surveys (*p* < 0.001) and increased somewhat between the second and third surveys, but not significantly different (*p* = 0.185). Even when the survey was complete, the KOSS-SF score was higher than the first survey’s results (*p* < 0.001). Additional analyses were performed by using adjusted model for age, tenure, gender, educational level, marital status, smoking status, alcohol consumption. In CES-D score, adjusted mean for first (7.66, 95% CI 5.97–9.36), second (11.03, 95% CI 8.92–13.14), and third (9.52 95% CI 7.57–11.47) survey showed similar trend but had no significant change over time (F[2, 400] = 0.466, *p* = 0.628). In post-hoc analysis, changes between first and second survey was statistically significant (*p* = 0.004) but changes between second and third survey, overall changes between first and third survey were not significant (*p* = 0.322, *p* = 0.175). Adjusted mean for KOSS-SF total score were 46.13 (95% CI 43.57–48.70), 50.43 (95% CI 48.14–52.72), 51.51(95% CI 48.94–54.08) each. The changes of adjusted KOSS-SF score was statistically significant (F[1.899, 379.831] = 3.192, *p* = 0.045) over time. Post-hoc analysis of KOSS-SF score showed same pattern compared to crude model (First-second: *p* = 0.001, Second-third: *p* = 0.999, First-third: *p* < 0.001).

**Table 2:** Mean CES-D score and KOSS-SF total score at 1st, 2nd, 3rd survey

Variables	1st Mean (SD) or Mean(95% C.I)	2nd Mean (SD) or Mean(95% C.I)	3rd Mean (SD) or Mean(95% C.I)	F	*P* ^*^	Post hoc^†^
CES-D Score	7.75(7.94)	10.42(10.06)	8.90(9.03)	9.255	< 0.001	A < B^‡^, B > C^‡^, A < C
Adjusted CES-D	7.66(5.97–9.36)	11.03(8.92–13.14)	9.52(7.57–11.47)	0.466	0.628	A < B^‡^, B > C, A < C
KOSS-SF score	45.03(12.23)	50.19(10.69)	51.50(11.93)	40.195	< 0.001	A < B^‡^, B < C, A < C^‡^
Adjusted KOSS-SF	46.13(43.57–48.70)	50.43(48.14–52.72)	51.51(48.94–54.08)	3.192	0.045	A < B^‡^, B < C, A < C^‡^

*A* 1st survey April 2014, *B* 2nd survey April 2015, *C* 3rd survey April 2016, *SD* Standard deviation, *C*. *I* Confidence interval

* *P* value was calculated by repeated measure ANOVA

^†^ By Bonferroni correction, ^‡^ *p* < 0.05

**Fig. 1:**
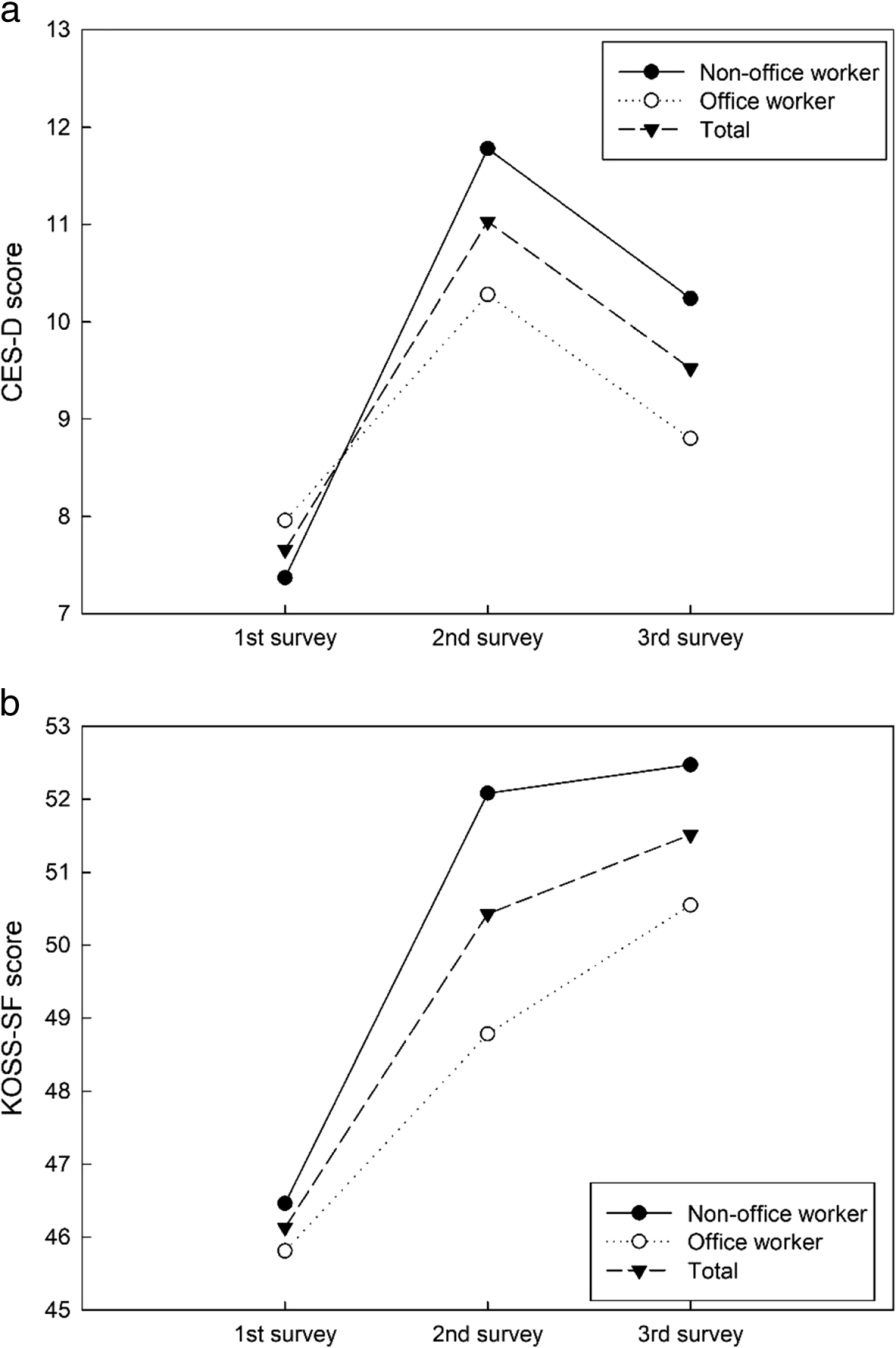
**a**: Comparison of CES-D score between non-office worker group and office-worker group, **b**: Comparison of KOSS-SF total score between non-office worker group and office-worker group

The differences in the CES-D score and KOSS-SF total score between the blue- and white-collar worker groups were analyzed by survey time via repeated measures ANOVA with adjusted model (Age, tenure, gender, educational level, marital status, smoking status, alcohol consumption). The analysis results are as follows. The difference in CES-D score between two groups was not significant statistically (between-group F = 0.316, *p* = 0.574). The interaction between survey time and occupational group was also not significant statistically (F = 0.967, *p* = 0.381). The difference in KOSS-SF total score between the two groups was not statistically significant (between-group F = 1.132, *p* = 0.289) and, the interaction between survey time and occupational group was also not statistically significant (F = 0.817, *p* = 0.437), as shown by the CES-D score. (Figure 1) indicates the adjusted changes in the CES-D score and KOSS-SF total score by survey time and occupational group. The initial CES-D score was the highest in the white collar group, followed by total workers, and then the blue collar group. This order was changed after all surveys were completed. Blue collar workers were the highest, followed by total workers and then white collar workers. The CES-D score was the highest at the second survey conducted after the merger and then tended to decrease afterward. The initial KOSS-SF total score was highest in the blue collar worker group followed by total workers and then the white collar worker group. The same order was maintained even after the survey was done. However, the KOSS-SF total score in the third survey increased somewhat even after the second survey conducted after the merger, in contrast with the CES-D score.

### Subgroup analysis of KOSS-SF

In subgroup analyses, Job insecurity and Lack of reward showed a significant difference over time (Job insecurity F[2, 400] = 8.025, *p* < 0.001; Lack of reward F[2, 400] = 3.296, *p* = 0.038; Table 3).

**Table 3:** Adjusted mean KOSS-SF subgroup score at 1st, 2nd, 3rd survey

Variables	1st Mean(95% C.I)	2nd Mean(95% C.I)	3rd Mean(95% C.I)	F	*P* ^*^	Post hoc^†^
Job demand	42.29(38.04–46.54)	40.31(36.82–43.79)	46.23(42.22–50.23)	0.423	0.646	A > B, B < C, A < C^‡^
Insufficient job control	55.58(51.59–59.57)	55.47(51.55–59.38)	56.61(53.11–60.11)	0.863	0.423	A > B, B < C, A < C
Interpersonal conflict	41.11(37.01–45.22)	37.33(33.34–41.32)	39.01(34.96–43.06)	0.199	0.808	A > B, B < C. A > C
Organizational system	51.41(47.54–55.27)	57.25(53.48–61.01)	58.27(54.44–62.10)	2.779	0.063	A < B^‡^, B < C, A < C^‡^
Lack of reward	50.50(46.26–54.74)	55.96(51.85–60.07)	60.43(56.29–64.57)	3.296	0.038	A < B^‡^, B < C, A < C
Occupational climate	40.63(36.74–44.52)	40.82(36.90–44.74)	44.34(40.14–48.55)	0.888	0.412	A < B, B < C, A < C
Job insecurity	37.71 ± 24.91	78.31 ± 25.26	60.29 ± 27.82	8.025	P < 0.001	A < B^‡^, B > C^‡^, A < C^‡^

*A* 1st survey April 2014, *B* 2nd survey April 2015, *C* 3rd survey April 2016, *C*. *I* Confidence interval

* *P* value was calculated by repeated measure ANOVA

^†^ By Bonferroni correction, ^‡^ *p* < 0.05

The results of the post-hoc analysis with Bonferroni correction showed that job demand, interpersonal conflict and insufficient job control scores decreased between the first and second surveys, then increased in the third survey. A significant difference in job demand was revealed in the second and third surveys (*p* = 0.008), whereas interpersonal conflict and insufficient job control had no statistically significant difference. The scores of the organizational system, lack of reward, and occupational climate subgroups tended to increase over time. But all subgroups except for lack of reward showed a no statistically significant difference by survey time. More specifically, lack of reward increased significantly in the first and second surveys (*p* = 0.038), but experienced no significant changes between the second and third surveys. The job insecurity subgroup showed a similar change, as aforementioned in the CES-D score (1st–2nd survey *p* < 0.001, 2nd–3rd survey *p* < 0.001), but significantly increased statistically even after the survey’s completion compared to the initial survey value (1st–3rd survey *p* < 0.001).

The differences in the subgroup scores of KOSS-SF between blue and white collar worker groups were as follows. The difference between blue and white collar worker groups (between-groups) was significant statistically for the job demand (*p* = 0.049), organizational system (*p* = 0.014) subgroups. The interaction between survey time and occupational group was not statistically significant in all subgroup.

## Discussion

This study aimed to verify whether there was a significant change in depression level and job stress in the process of restructuring after a merger without massive downsizing according to survey administration time. It also aimed to determine whether there was a difference between blue and white-collar worker groups during this significant change, and whether there was an interaction between occupational group and survey time.

There have been controversial debates in previous studies on the effects of mergers on mental health. This study revealed that the adjusted level of depression by age, tenure, gender, marital status, smoking status, alcohol consumption found through the CES-D score had changed but were not statistically significant over time and the level of job stress surveyed through the KOSS-SF total score was significantly different according to the survey time. These results are similar with the Netterstrøm et al. (2010) [23], who found that a merger without downsizing did not result in a significant change in depression risk, increase in depression reported by Isaksson et al. (2000) [17], the increase in job stress reported by Woodward et al. (2000) [34], and the increase in job stress reported by Brown et al. (2006) [22]. However, these results differ from those obtained by Vahtera et al. (1997) [27], who found that being laid off was the most influential factor, Kokkinen et al. (2013) [28], who found that the probability of long-term sick leave did not increase significantly when merger was not accompanied by a lay-off, and increase in depression reported by Isaksson et al. (2000) [17]. Since only some of the previous studies on merger without accompanying downsizing obtained different results, it is difficult to understand what the differences between these previous studies and this study are, but the results of this study can be explained as follows.

First, the results of this study are consistent with those presented by Falkenberg et al. (2013) [25], who found that harmful health effects were generated through increased stress and job instability, and van der Ploeg. et al. (2003) [35], who presented an explanation using the model that chronic stress factors, such as job instability, and acute stress factors, such as unemployment, can develop into symptoms through the post-traumatic reaction, and during this process, chronic stress factors directly affect the development of these symptoms. Some of the previous studies [23, 28] found that acute and chronic stress did not increase without lay-offs. However, cultural differences between the countries these studies were conducted in and Korea should be taken into consideration. After the merger, the study’s target business became a relatively small-sized company. According to a sociological study [36], Korean people have higher social pressure to demonstrate status than in other countries, and inferior material or social status can seem like personal inferiority. Thus, the downsizing of the company may be a stress factor. In addition, a study by Do (2005) [37] said that specific organizational cultural traits could affect organizational effectiveness, and as Korea has a peculiar enterprise culture regarding the merger of a company, worry about the changes could play a role as a stress factor, as explained in a study by Suh (2010) [38]. Finally, a worker’s union was formed for the first time in the study target business in the middle of the merger process, and labor disputes continued even after the merger. A study [39] surveying job stress during a labor dispute using the full version of KOSS also reported a similar increase in the KOSS total score. Thus, the labor disputes accompanied by a merger in this study acted as a stress factor, thereby increasing job stress.

One interesting fact is that the levels of depression and job stress changed significantly over time, but the pattern of change was different. As shown in Fig. 1, a difference in the pattern showed that the CES-D score decreased over time as a short-term effect, as presented in a study by Dahl (2011) [40], whereas the KOSS-SF total score was maintained for a certain period or increased as a long-term effect, as reported in other previous studies [25, 28, 41]. Zapf (1996) [42] explained that differences were due to uncertainty associated with a changer over time. This may be also because the KOSS-SF total score was calculated by dividing the sum of the subgroup results with different characteristics by the number of subgroups. During the survey, employees often said that physical/non-physical work load was decreased during a merger process accompanied by lack of supervision and these mentions were supported by change patterns of job demand, insufficient job control and interpersonal conflict. Finally, mechanisms between a depression and job stress increase are different by nature. Future study should be focused to understand more possible mechanism of these differences.

The CES-D scores and KOSS-SF score for blue and white collar worker groups did not reveal any difference between these groups, and no meaningful interaction was exhibited between the occupational groups and survey administration time. In subgroup analyses, no statistically significant interactions were found in all subgroup. These results showed that mental health outcome after merger seldom affected by traditional work group difference for blue and white collar workers.

The limitations of this study are as follows: first, the mean age and work tenure of the subjects in the first survey were respectively 26.52 ± 3.78 and 6.22 ± 3.87 years, which limited the study to relatively young workers who had a short work tenure. These limitations were the result of the subject selection process. In this factory, employees over 40 years are able to choose more comprehensive medical examination in different time point (usually in July to October) every other year. Thus, the influences [29] of previously known job stress-related factors, such as age or work tenure, may be underestimated, which makes it difficult to generalize the study results to all working populations. Because repeated measure-ANOVA was used as a method in research design process, the subjects with the different survey time-point were strictly excluded. In future study, statistical methods that is possible to handle missing values and different survey time-range such as the generalized estimating equations (GEE) will be the solution for this generalization problem.

Second, significant differences occurred in the baseline general characteristics that were inherent in the occupational group itself when classifying the blue and white collar worker groups. A statistically significant difference was revealed for gender, smoking status, and high risk drinking status in addition to the aforementioned age and work tenure. Even after these variables were adjusted as covariates, there can be possibility of unidentified confounders. Therefore, study designed by more advanced statistical methods such as, mixed effect model (MEM) or generalized estimating equation (GEE) should be established to correct confounding variables that may mask the effect of the differences in the occupational group on depression and job stress. Despite the above shortcomings, this study contributed to the observation of changes in depression and job stress in the middle of a merger without an accompanying mass lay-off using a scaled tool in Korea and increased test power by performing repeated measures ANOVA and pairing the same subjects in the same survey period.

## Conclusion

This study determined that the merger without an accompanying massive downsizing can cause negative health effects, such as an increase in depression and job stress. In addition, even these adverse effects can be different because both short- and long-term effects were found in the difference between the change patterns of depression and job stress. Thus, to improve the health of workers, both the negative effects themselves and the resolution or maintenance of effects on health over time should be considered prior to a company merger. These considerations are factors that should be considered when establishing a management or stress prevention program in a company. For a future study, more controlled study subjects are needed for the clear identification of the effects according to the differences in occupational groups, and subjects from a more general population to ensure that the results of this study are applicable to a general working population.

## Abbreviations

ANOVA: Analysis of variance
C.I: Confidence interval
CES-D: The Center for Epidemiologic Studies Depression Scale
IMF: International Monetary Fund
KOSS-SF: The Korean Occupational Stress Scale-Short Form
N: Number
SD: Standard deviation
